# Development of a Nanoscaled Ion Source for High-Sensitivity Photoionization Mass Spectrometry

**DOI:** 10.1021/acs.analchem.5c06912

**Published:** 2026-04-10

**Authors:** Laura Tenhumberg, Wolfgang Schrader, Alessandro Vetere

**Affiliations:** 28314 Max-Planck-Institut für Kohlenforschung, Kaiser-Wilhelm-Platz 1, 45470 Mülheim a. d. Ruhr, Germany

## Abstract

Atmospheric Pressure Photoionization (APPI) has emerged as a versatile ionization method in mass spectrometry, able to ionize compounds of comparably low ionization potential (typically <10.6 eV), irrespective of their functionalities. Aromatic analytes are particularly well suited for photoionization because their delocalized π-electron systems result in comparatively low ionization potentials, enabling both direct single-photon ionization and ionization by charge transfer from dopant ions. While highly effective for a broad range of analytes, APPI faces issues of high sample consumption and elevated background noise, which can limit its effectiveness in trace analysis. Prior advances in miniaturizing flow rates, as seen with the transition from conventional Electrospray to nano-Electrospray, have shown that reducing flow rates not only conserves sample but can also enhances sensitivity by increasing the signal-to-noise ratio. Applying similar miniaturization strategies to APPI holds promise for overcoming current limitations and improving its analytical performance. This study investigates the impact of lowered analyte concentrations and reduced flow rates on the sensitivity of APPI for ultratrace analysis. We introduce a prototype ion source for APPI applications and systematically explore the effects of flow rates below 1 μL min^–1^ on APPI performance, evaluating the signal-to-noise ratios and detection limits achieved. Our findings indicate that reduced flow rates significantly improve sensitivity, demonstrating the potential to detect ultratrace levels of environmental pollutants with higher efficiency and lower background interference.

## Graphical abstract

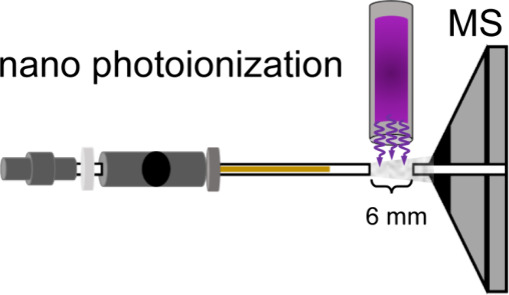

## Introduction

Among the key factors contributing to the widespread adoption of mass spectrometry are its ability to perform both qualitative and quantitative analyses, its compatibility with chromatography for enhanced separation, its capability for structural elucidation, and its ability to analyze multiple components at the same time. The continued development of mass spectrometric techniques has been driven by the need for accurate, efficient analysis of increasingly complex samples and the demand for lower detection limits. Central to this progress are the ionization techniques as part of a mass spectrometric measurement, which are crucial for converting analytes into gas-phase ions. Each technique is specifically suited for different types of compounds based on their chemical properties, such as size and polarity, enabling a wide range of applications.^1–7^

Among the various ionization methods, Atmospheric Pressure Ionization (API) techniques have become particularly popular due to their gentle ionization processes. These methods allow for minimal fragmentation, thus preserving the integrity of the analyte. Atmospheric Pressure Photoionization (APPI) was developed in 2000 to provide an ionization technique for medium to nonpolar and aromatic compounds that were difficult to ionize using API methods like Electrospray Ionization (ESI) or Atmospheric Pressure Chemical Ionization (APCI).^8,9^ Unlike ESI, which relies on an electric field to create charged droplets,^10^ APPI uses vacuum ultraviolet (VUV) radiation, typically from a krypton discharge lamp (photon emission at 10.0 and 10.6 eV) to photoionize analytes with low ionization potentials (IP) such as polycyclic aromatic hydrocarbons and polycyclic aromatic heterocycles (PAH and PAXH), leading to radical cations:

1 $$M+h\nu \to M^{•+}+e^{-}$$

As only singly charged ions are formed, the method is particularly suitable for small to moderately sized molecules, with a mass range typically up to 1000 Da.^8,11,12^

While other light sources (e.g., Ar- (11.2 eV) or Xe-lamps (8.4 eV)) do exist, Kr-lamps constitute a good compromise between universality (many compounds have an IP < 10.6 eV) and selectivity (common solvents, such as methanol (IP = 10.84 eV) are not ionized by a Kr-lamp).^13^ Though not ionized at these energy levels, most matrix components, including water and molecular oxygen, have a considerable absorption cross section for the photons.^14^ This leads to excitation and eventually also to photodissociation of many components, resulting in two undesired side-effects:

1) Excitation and/or photodissociation of compounds like water, methanol or oxygen leads to the presence of reactive species (atomic oxygen, OH^•^, CH_3_^•^, CH_3_O^•^) that promote oxidation of analyte molecules/ions and^15^
2) the absorption of photons leads to an exponential decay of light intensity with growing distance from the light source, i.e., the availability of photons for direct photoionization of low abundant analytes is generally poor.

APPI is carried out on a gas-phase sample. To this extent, the liquid sample solution is introduced into the ion source through a heated pneumatic sprayer, i. e., the sample is injected into a heated zone (typically >300 °C), where the bulk solvent is rapidly evaporated. The gaseous sample mixture is then transported into the ionization region – the zone irradiated by the Kr-lamp – by a constant carrier gas stream (typically N_2_) that also helps in nebulizing and further desolvating the sample (see also Figure S1 in the Supporting Information for a schematic view of an APPI source).

The photoionization probability of an analyte molecule in the resulting aerosol is typically very low as it is only present in small amounts and most photons are depleted by matrix constituents, such as the solvent.

The overall ion yield can be improved by increasing the particle density of a readily ionizable compound within the sample aerosol. This approach has been termed Dopant-Assisted APPI (DA-APPI or DAPPI).^8^

When (partly) replacing a nonionizable solvent by a readily ionizable dopant (D) – e.g. toluene (IP = 8.83 eV) – a reasonable amount of initial photoionized particles can be created. At sufficiently high particle densities, collisions between these ions, analytes and/or other matrix components occur frequently. This switches the ionization mechanism from direct photoionization to a type of chemical ionization. This route is promoted through secondary ion–molecule reactions with the formerly created dopant ions, while under these circumstances direct photoionization of the analytes hardly plays any role.

Different subsequent reaction pathways are possible, depending on the solvent mixture and the type of analyte, which can ultimately lead to an increased amount of available analyte ions.

For a solvent or analyte molecule M with IP­(M) < IP­(D), direct charge transfer (CT) may occur, leading to a radical cation:^16^

2 $$D^{•+}+M\to D+M^{•+}$$

For analytes (M) with a proton affinity PA­(M) > PA­([D-H]) (in case of toluene as dopant, [D-H] is the benzyl radical), proton transfer (PT) may occur:^16^

3 $$D^{•+}+M\to {\left[D-H\right]}^{•}+{\left[M+H\right]}^{+}$$

It has also been proposed that protonation of the analyte by dopant ions might proceed via charge transfer (eq 2), followed by hydrogen transfer within the intermediate analyte-dopant-complex.^17^

When methanol and/or water are present in the solvent mixture, such proton transfer leads to the efficient depletion of dopant ions from the aerosol and the generation of protonated water/solvent clusters, which in turn can serve as reagents for the subsequent protonation of an analyte molecule:^11,18,19^

4 $$\begin{array}{l} D^{•+}+nH_{2}O\to {\left[D-H\right]}^{•}+{\left[H+{\left(H_{2}O\right)}_{m}\right]}^{+}+\left(n-m\right)H_{2}O \end{array}$$

5 $${\left[H+{\left(H_{2}O\right)}_{m}\right]}^{+}+M\to {\left[M+H\right]}^{+}+{\mathrm{mH}}_{2}O$$

Equations 4 and 5 have been simplified here for brevity. The pathway by which an analyte ion will be formed, strongly depends on the nature of the matrix components, their relative abundances in the source region and the overall particle density. Possible ways to help keep the signal of matrix background low and that of the analytes up, are the reduction of compounds that lower the photon penetration depth (e.g., oxygen, water) and a reduced overall particle density.

Conventional APPI, as it is commercially available today, operates at solvent flow rates of 50 to 200 μL min^–1^. While this constitutes a rather high sample consumption when compared to electrospray (3–10 μL min^–1^) it also often leads to challenges such as clogging and increased maintenance efforts. It has already been shown that further increasing these flow rates has detrimental effects on the ion generation and signal intensity.^20^ Increased flow rates lead to elevated particle densities of nonionizable solvent molecules in the ionization region (i.e., a solvent that cannot be directly ionized by interaction with photons of 10.0/10.6 eV), which in turn reduces the penetration depth of photons into the sample aerosol. Although protonated solvent clusters play an important role in the formation of protonated analyte molecules in DA-APPI, this ultimately leads to signal depletion. In addition to solvent-related limitations, the geometric design of commercial APPI sources has also been identified as a critical factor influencing ionization efficiency. In comparison to commercially available “open geometry” designs, McCulloch et al. demonstrated that an orthogonal APPI configuration featuring a defined, enclosed field-free reaction region that has a closely positioned VUV lamp can provide up to an order-of-magnitude improvement in sensitivity.^21^

Over the years, mass spectrometry has seen numerous innovations on ion source designs and analyzer developments, with manufacturers commercializing these advancements. Notably, miniaturization breakthroughs in ionization techniques, with nano-ESI as a pioneering development in 1994 by M. Wilm and M. Mann, have become widely used in the field. The availability of nano-ESI led to the adoption of nano-HPLC as separation method for a whole variety of polar compounds. Similar methods for nonpolar compounds are not available, probably due to missing equivalent nano sources. Nanoflow applications in APPI are not compatible with the currently available commercial sources. It has already been shown that lowering the flow rate in a conventional APPI source beyond a few dozen microliters per minute results in a loss of signal.^20^ This can be attributed to an insufficient mass flow of dopant as well as analyte, i.e., the amount of produced ions is so low that a) secondary ion–molecule reactions to form analytes via the dopant-assisted pathway become improbable and/or b) loss of ions through recombination or neutralization at source walls becomes dominant. Therefore, any attempt to reduce the applicable flow rates in APPI to the nanoliter per minute level, should include a redesign of the conventional, available sources.

In the early 2000s, lab-on-a-chip designs became popular. These microfluidic systems integrate chemical or biological analysis on a miniaturized chip. Microchip-APPI applied this principle, combining microfluidic channels with a heated nebulizer to enable efficient photoionization at low flow rates in the low μL min^–1^ range. The first microchip-based APPI, developed by Kostiainen and co-workers, featured a microstructured heated nebulizer using a krypton discharge lamp for photoionization. This design, with flow rates between 0.05 and 5 μL min^–1^, proved to be a stable and efficient alternative.^22^ Similar designs followed, including applications for FT-ICR MS analysis of highly complex petroleum samples. By reducing flow rates 25 times compared to conventional APPI, these systems offered a significant improvement, reducing contamination and sample usage, albeit with a 40% loss in signal response.^23^ A few more microchip designs followed, and the coupling with chromatographic systems was also tested.^24–31^ Disadvantages remain to this day, such as the complexity of manufacturing and integrating various functions on a single chip.

In this study, we aim to pursue a different approach by developing a miniaturized nano-APPI (nAPPI) source. Key aspects of the new design will be the miniaturization of the entire ionization region to make it compatible with the necessities of low flow rates. This also includes a largely reduced distance of the sample aerosol to the VUV lamp. Together with an envisioned lower particle density, this should increase photon penetration depth and minimize the effect of residual water/oxygen in the surrounding atmosphere. While we anticipate that the overall signal intensity may decrease due to the lowered sample introduction, we propose an improved ionization efficiency and, thus, an increasing signal-to-noise ratio (S/N), resulting in better sensitivity at reduced flow rates. The goal is to demonstrate the feasibility and advantages of nAPPI for practical applications, showcasing its potential to broaden the landscape of mass spectrometry and analytical technologies.

## Experimental Section

### Ion Source Design

In this study, a conventional APPI source was reconstructed and converted into a miniaturized format (Figure 1). Thus, the aim is to retain a modular setup that enables simple part replacement and also allows for swift disassembly for cleaning and maintenance purposes. Specifically, the sprayer is modified by replacing the common ceramic heater with a 16-gauge stainless steel capillary that serves as the heating zone, reducing the cross sectional area by 95% compared to the corresponding conventional APPI source, which offers an improved response time of just a few milliseconds. As sample carrier, a fused silica capillary of 50 μm inner diameter is slotted into the heater capillary. Compared to conventional APPI (150 μm) this amounts to a reduction in the cross sectional area by 90%. Unlike lap-on-a-chip-based designs, which require very small heating elements, a copper block equipped with a 200 W heating cartridge is chosen, which provides excellent thermal conductivity. It allows to operate near the original APPI heating levels (250 W) and, thus, making use of the mass spectrometers built-in heating circuitry. While temperature settling times are slightly higher than for conventional APPI due to increased thermal mass, the temperature stability is excellent, once it has been reached (Figure S2). Due to the reduced sample flow rates and overall dimensions, the setup can be operated at relatively low desolvation temperatures of 120 to 200 °C as compared to conventional APPI, where temperatures often exceed 350 °C.

**Figure 1:**
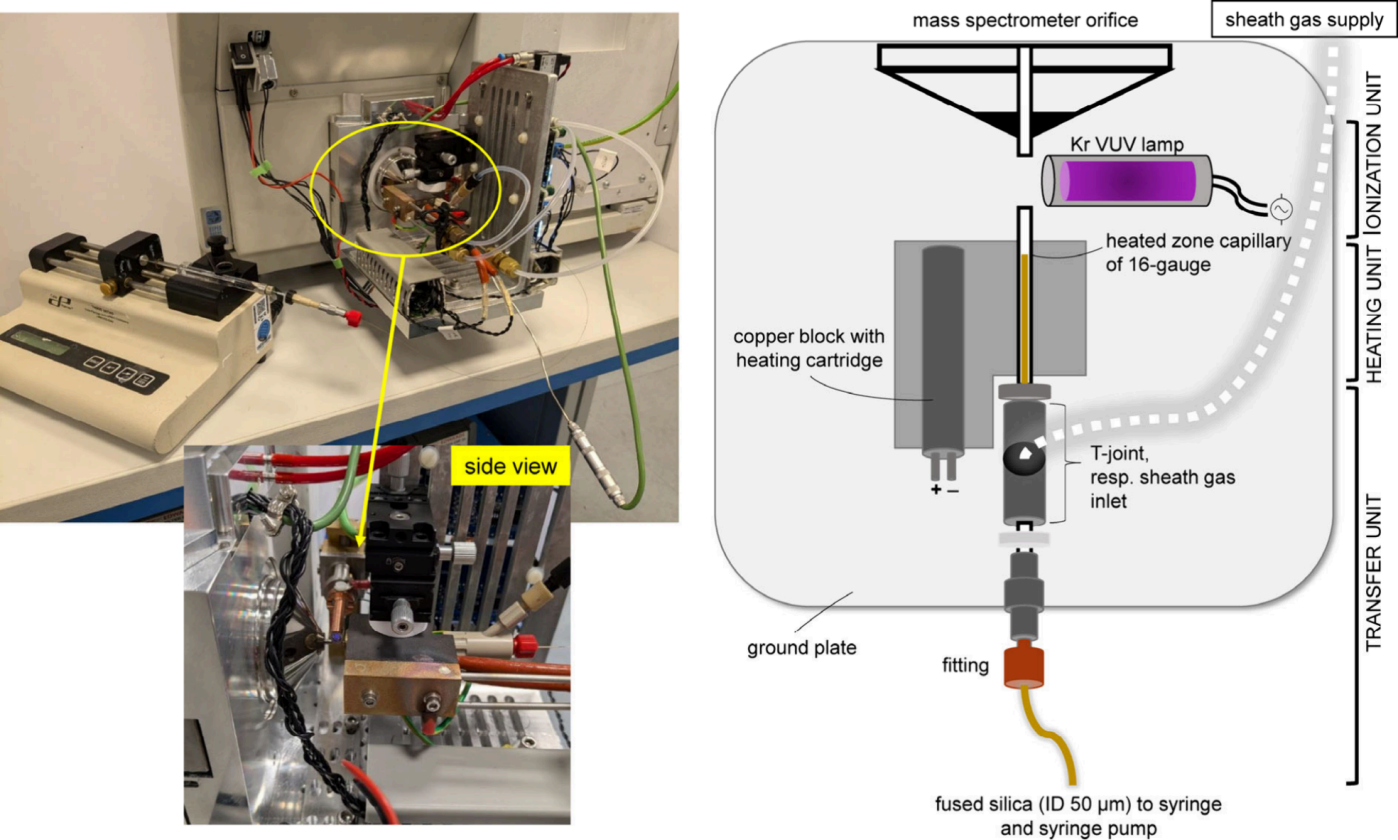
Photo of the nAPPI source coupled to a TSQ triple quadrupole MS (left) and schematics (right).

Due to the reduced flow rates required for micro- to nanoflow applications, a coaxial alignment between the nAPPI sprayer and the mass spectrometer orifice is utilized (Figure S1). To overcome space limitations, the Kr VUV lamp used for conventional APPI (Syagen Technology Inc., Tustin, CA, USA, 12.7 mm bulb diameter, excitation via a 13.56 MHz RF-coil) is replaced with a smaller one of only 6 mm diameter (Heraeus Noblelight GmbH, Hanau, Germany, RF-excited at 100 kHz). To further optimize space requirements, the excitation circuit has been customized to remove bulky plastic components from the heated area around the MS orifice. Additionally, the electrode setup has been changed from a pair of coaxially placed electrodes through which the bulb is slotted to a pair of radially placed electrodes around the bulb, covering a radial sector of 90° each (see also Figure S3). The cross sectional area of the lamp is reduced by 78%. This allows us maintaining a smaller distance between the sprayer nozzle and the mass spectrometer, while providing enough space for the lamp to avoid thermal damage from heat. This design reduces the size of the ionization zone from 3000 mm^3^ for conventional APPI to around 200 mm^3^ (Figure S1). The ionization process is further optimized through the use of a heated nitrogen sheath gas, which helps to finely nebulizing the analytes. We have introduced a proportional valve to reduce the sheath gas flow to 80 mL min^–1^, which is a reduction by 73–95% of the original APPI gas flow, while maintaining efficient nebulization and minimizing gas consumption. Initial attempts to profit from an enclosed ionization region, similar to the works of McCulloch et al., remained unsuccessful due to a mechanic instability of the miniaturized parts and have therefore been discarded.

Designed to operate at nanoelectrospray-like flow rates of 500 nL min^–1^ or below, nAPPI not only minimizes sample consumption but also significantly reduces contamination risks and simplifies maintenance and cleaning as all wetted parts are made from easily cleanable materials such as stainless steel.

Additionally, the system’s modular design with replaceable consumable components and a removable sprayer ensures sustainability and reusability, making it an efficient solution for long-term analyses.

### Mass Spectrometry

In a first iteration, the nAPPI source is designed to be compatible with various mass spectrometers using the IonMax source from Thermo Fisher Scientific (San Jose, CA, USA). Optimization experiments were conducted on a TSQ Quantum Ultra AM (Thermo Fisher Scientific, San Jose, CA, USA). Further measurements were performed on an LTQ FT Ultra FT-ICR MS (7T, Thermo Fisher, Bremen, Germany). Measurements were evaluated using Thermo Xcalibur software (v. 4.2, Thermo Electron, Bremen, Germany).

During testing, full-scan measurements were recorded for 2 min for each parameter to optimize the source. Phenanthrene (Sigma-Aldrich) was used as a reference analyte, and a stock solution of 1 g L^–1^ in toluene (Merck) was prepared. For iteration experiments dilution series were created down to 1 ng L^–1^, and signal-to-noise (S/N) ratios were calculated for each experiment. The mass range of 50–500 Da was recorded to evaluate the quality of the measurements. Besides phenanthrene, a set of different PAH/PAXH and further pharmaceutically relevant compounds were tested (Table S1).

To evaluate S/N ratios for phenanthrene, fullscan measurements of a constant solvent flow (50 μL min^–1^ for APPI and 1 μL min^1^ for nAPPI) were used. Into this stream, solutions of concentrations between 1 ng L^–1^ and 100 mg L^–1^ were injected through a sample loop (50 μL for APPI and 2 μL (filled with 1 μL analyte solution) for nAPPI). From the recorded mass spectrum the extracted ion chromatogram (XIC) of the molecular ion (*m*/*z* 177.5–178.5) was used for S/N calculations by using the mean signal intensity before and after the injection peak as noise level and the injection peak intensity as signal value.

The Limit of Detection (LoD) was determined by plotting the resulting S/N ratios against the analyte concentration/mass flow. At low concentrations (1 ng L^–1^ to 10 μg L^–1^) the resulting S/N behaves linearly and can be extrapolated to find the concentration/mass flow, were S/N = 3. This value is considered the LoD.

To demonstrate the real-world applicability of nAPPI also for complex samples, a heavy crude oil sample was diluted to 300 mg L^–1^ and analyzed by FT-ICR MS. Its ionization efficiency was evaluated against conventional APPI and chemical formulas annotation was performed using Composer software (v. 1.5.6, Sierra Analytics, Modesto, CA, USA) within the following constraints:

$$C_{0-200}H_{0-1000}N_{0-2}O_{0-4}S_{0-3},-0.5<\mathrm{RDBE}<100.0,\mathrm{mass error}<1.2\mathrm{ppm}$$

## Results and Discussion

### Addressing High Background Noises in Photoionization

The choice of solvent in mass spectrometric analyses is influenced by several factors, including the mass of both the solvent and the analyte. Ideally, a solvent that does not ionize itself is preferred. Many common solvents such as methanol or acetonitrile have ionization potentials above 10.6 eV and are thus not ionized by APPI. However, for some problems, like the analysis of polycyclic aromatic hydrocarbons (PAH) in complex mixtures, the addition of solvents such as toluene might be necessary for solubility reasons. In such a case, ensuring a clear distinction between the solvent and analyte masses becomes crucial. This is because the solvent typically constitutes the main fraction of the spray, generating strong signals that may suppress analyte ionization and reduce the S/N. To obtain optimal results, it is often necessary to exclude the mass range dominated by solvent signals.

In a pure toluene blank spectrum obtained with APPI in-source reactions such as oxidation and oligomerization of toluene are often observed, leading to the formation of numerous interfering signals (Figure 2, top traces). The signals are in good agreement with reports from the literature.^32^ For a high-resolution spectrum with assignments of the most prominent signals, please refer to the Supporting Information, Figure S4. These signals often lead to the mass range below *m*/*z* 250 being of limited value. In nAPPI, only the toluene radical cation and a single signal at *m*/*z* 108 – corresponding to the incorporation of one oxygen, forming oxidized toluene – are observed (Figure 2, bottom traces). While the presence of the oxidation product suggests that oxygen and/or water are still present in the ionization region, their relative abundance is reduced, presumably by closing the gap between lamp window and sample aerosol. The mass range above *m*/*z* 108 is entirely free of additional oxidation or oligomerization products, demonstrating a remarkable reduction in background signals. This can be attributed to the overall smaller density of ionizable particles, in this case toluene. While the volume of the relevant ionization region has been reduced by a factor of around 15, the sample flow rate was reduced by a factor of 50–200. Thus, secondary reactions of toluene radical cations with excess toluene molecules and/or O_2_/H_2_O are minimized. When measuring phenanthrene in toluene using nAPPI, the resulting mass spectra are consistently clean and show a well-defined phenanthrene signal across a broad concentration range. A high long-term stability and good reproducibility are recorded for nAPPI (Figure S5).

**Figure 2:**
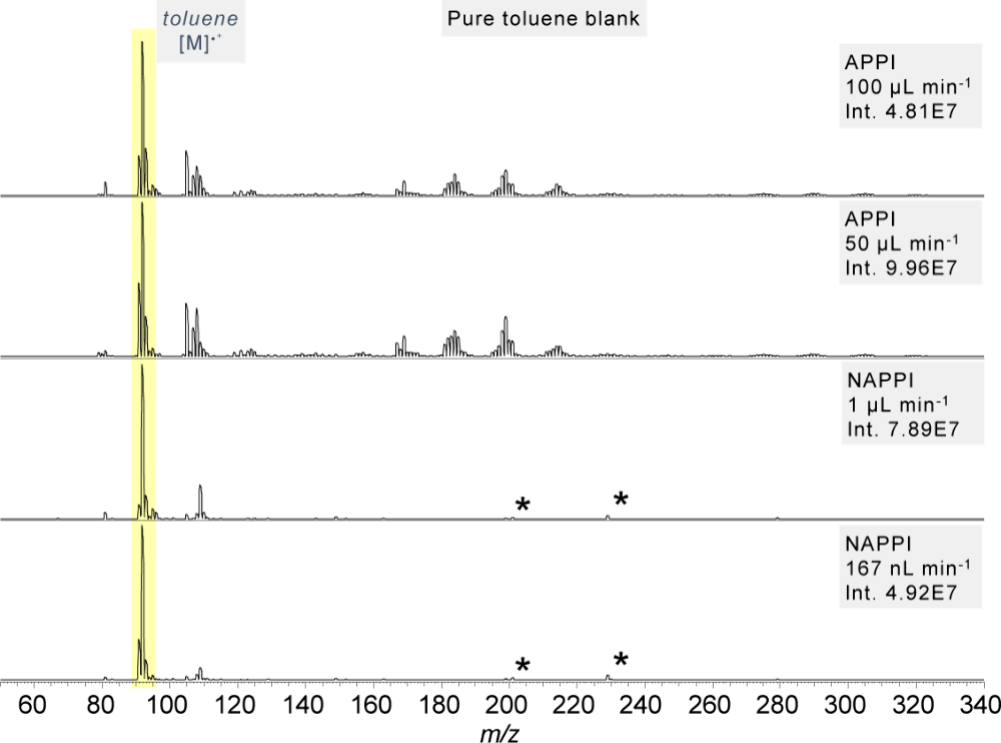
Pure toluene blanks for APPI and nAPPI at different flow rates. *Plasticizer impurities.

Notably, even at a concentration of 100 μg L^–1^ and a flow rate of 1 μL min^–1^, the molecular ion of phenanthrene is detectable, indicating a high level of sensitivity. As the concentration increases, the intensity of the signal scales accordingly, while background noise remains low and stable. The same trends can also be observed over a wide flow rate range (Figure S6). This results in distinctly clearer spectra compared to conventional APPI, particularly with respect to the S/N, which improves substantially with nAPPI (Figure 3). The clean spectral appearance and robust response demonstrate the effectiveness of nAPPI for the ionization of nonpolar aromatic hydrocarbons in organic solvents like toluene. These findings support the method’s applicability where a high S/N is critical for confident compound identification.

**Figure 3:**
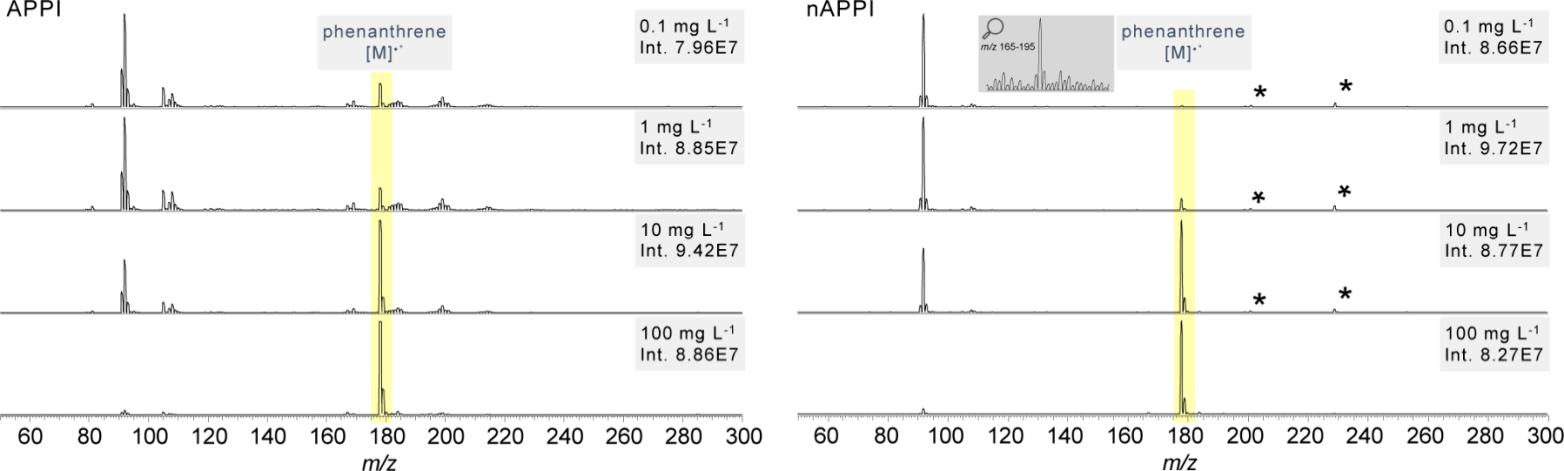
Measurements of phenanthrene in toluene at different concentrations with APPI at a flow rate of 50 μL min^–1^ and nAPPI at a flow rate of only 1 μL min^–1^. All spectra were measured on a TSQ triple quadrupole MS. *Plasticizer impurities.

Since many other analytes that are efficiently analyzed by APPI, including the entire EPA list of '16 PAH Priority Pollutants', fall within the mass range below 300 Da, the APPI background signals can severely hinder the detection of trace-level compounds. This is particularly problematic for PAH and PAXH, which are frequently studied environmental pollutants and require sensitive and selective detection methods. These compounds are highly soluble in toluene, which also serves as an effective dopant for photoionization. Figure 4 presents spectra of various PAH and PAXH in toluene obtained with both APPI and nAPPI. Although the flow rate is reduced by a factor of 100 when switching from APPI (100 μL min^–1^) to nAPPI (1 μL min^–1^), the resulting spectra remain highly comparable, while with a reduction factor of nearly 600 all analytes are still clearly observable. Notably, the signal intensities of carbazole, phenanthrene, dibenzothiophene, and 2-cyclohexylethanobenzothiophene are higher in nAPPI than in APPI, resulting in markedly improved S/N ratios for these analytes. The relative proportions of the two dominant signals, 3-methylquinoline and benzo­[*a*]­pyrene, are preserved in nAPPI, demonstrating the consistency between the techniques. Within the nanoflow regime, which cannot be accessed with conventional APPI due to hardware limitations, all analytes remain detectable with nAPPI. These findings highlight the clear advantages of nAPPI for applications involving limited sample amounts or extended acquisition times.

**Figure 4:**
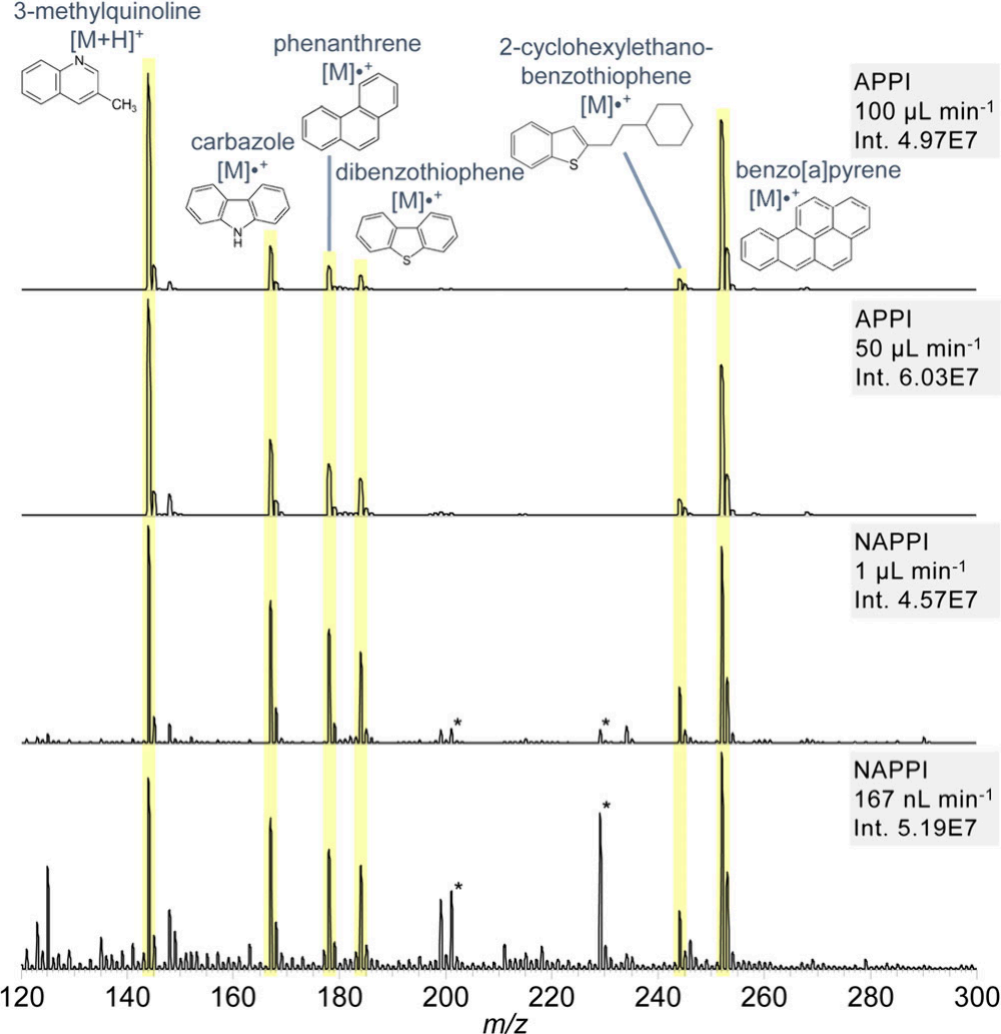
PAH/PAXH, 10 mg L^–1^ each in toluene, measured with APPI and nAPPI at different flow rates (measured on a TSQ triple quadrupole MS). *Plasticizer impurities.

### Improvement in Signal-to-Noise Ratio

The S/N is a key metric in analytical performance. A higher ratio indicates improved detection capability and reduced interference from noise. As previously noted, toluene, when used as solvent in conventional APPI, generates significant background noise due to the high toluene particle density, promoting oxidation and oligomerization reactions. This makes it difficult to separate analyte signals from this interference. Achieving a high S/N is particularly crucial when detecting trace concentrations, as even minor background signals can obscure analytes, resulting in inaccurate or unreliable measurements. Figure 5 illustrates the S/N calculations for both nAPPI and APPI. Concentration based values are slightly lower for nAPPI but generally comparable for the same sample concentrations (top *x*-axis) but differ largely when considering the corresponding flow rates (see analyte mass flow on the bottom *x*-axis). With increasing analyte mass flow, the S/N ratio rises for both ionization sources, with the effect being much more pronounced for nAPPI, resulting in higher S/N ratios at high concentrations (10 mg L^–1^ and 100 mg L^–1^ phenanthrene in toluene).

**Figure 5:**
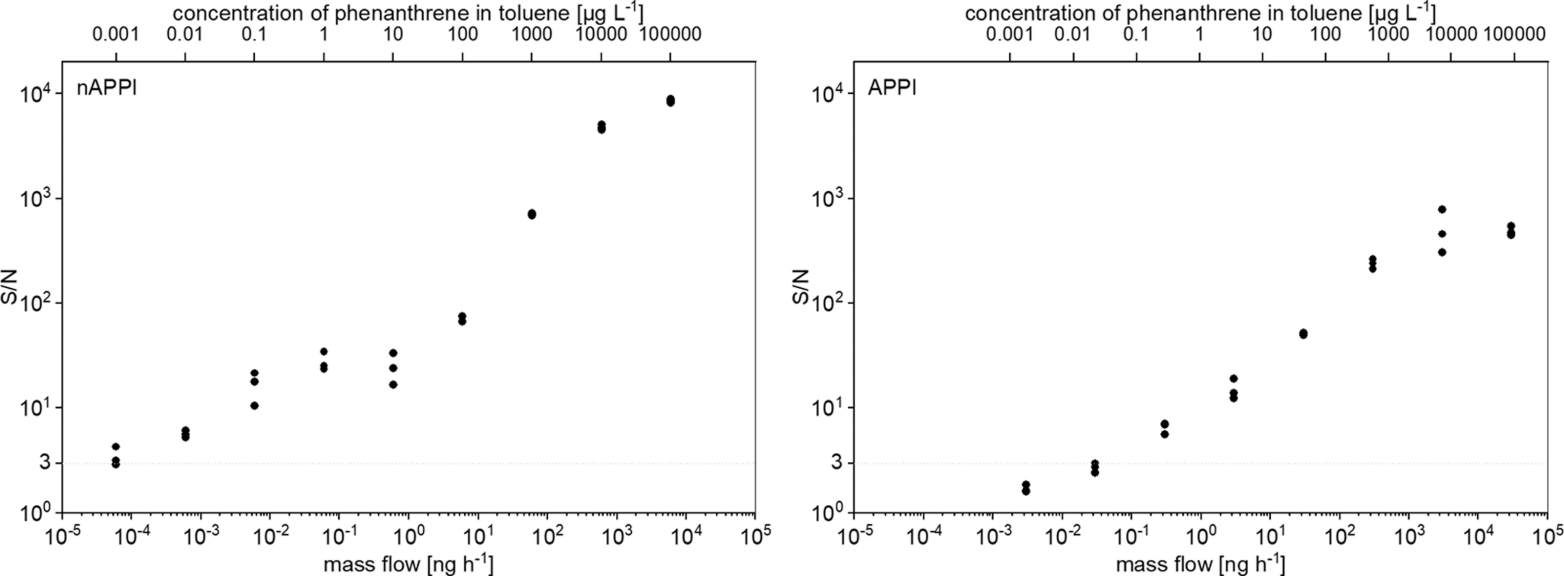
S/N calculations for nAPPI (flow rate of 1 μL min^–1^) and conventional APPI (flow rate of 50 μL min^–1^).

At very low phenanthrene concentrations differences between the methods become apparent. While with nAPPI the S/N deceases predominantly due to a decreased analyte signal, with APPI it is mostly due to an increased fluctuation of background noise that the S/N is lowered (Figure 6).

**Figure 6:**
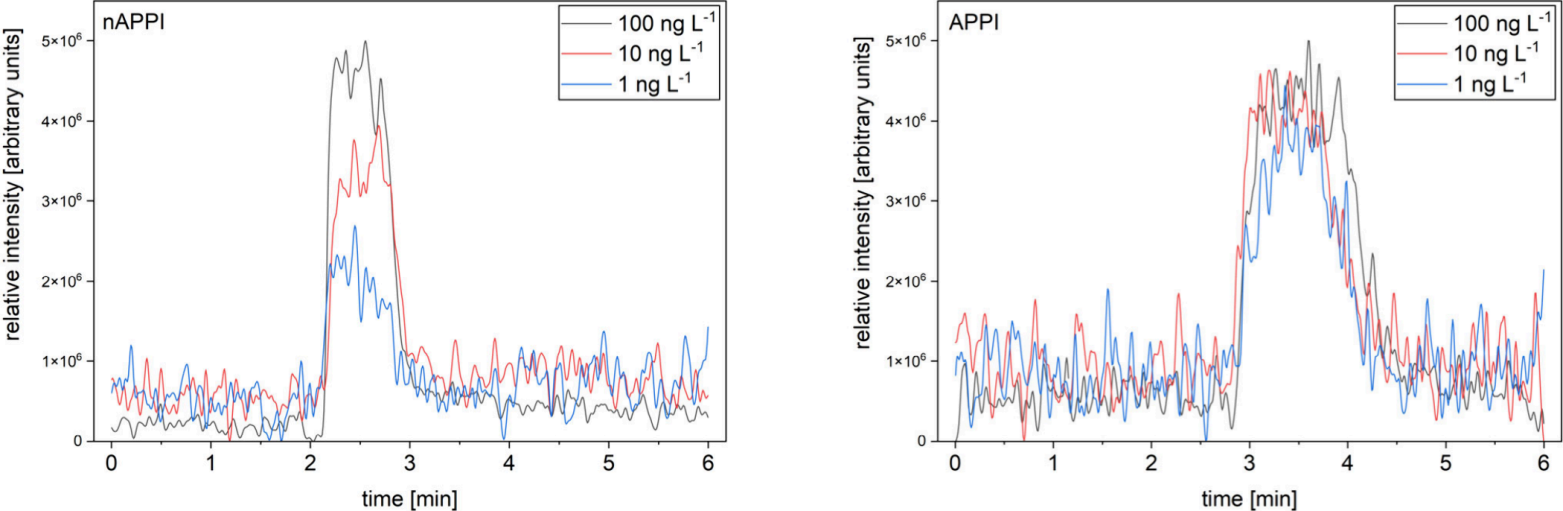
Mass traces of loop injections of phenanthrene in toluene at low concentrations for nAPPI and APPI.

### The Limit of Detection

The increased sensitivity directly leads to a decreasing LoD, which is the lowest concentration of an analyte that can be reliably distinguished from background noise or a blank. The LoD for phenanthrene is exceeded at a concentration of 1.10 × 10^–3^ μg L^–1^ for nAPPI and 7.62 × 10^–3^ μg L^–1^ for APPI. Taking into account the different flow rates, this results in an analyte mass flow of 6.62 × 10^–5^ ng h^–1^ for nAPPI and of 2.29 × 10^–2^ ng h^–1^ for APPI. Thus, the nAPPI source provides an improvement by a factor of 346 (by mass flow) in the LoD compared to conventional APPI. With respect to mass concentrations the improvement in LoD is 7-fold. The reduction in mass flow, and therefore particle density, and the smaller ionization region in nAPPI result in a better ionization efficiency with a reduced impact from matrix background components. This contributes to its superior sensitivity, enabling the detection of trace analytes at much lower mass flow rates. The enhanced sensitivity is particularly valuable in applications requiring sensitive detection of environmental pollutants, trace drugs, or other low-concentrated analytes. The overall reduction in mass flow will also prove beneficial in cases, where the available amount of sample is limited. Additionally, by minimizing ion suppression and improving the S/N, nAPPI allows for more reliable and accurate measurements in challenging analytical scenarios.

### Alternative nAPPI Analytes

To further evaluate the ionization efficiency of nAPPI, we analyzed a set of structurally diverse compounds spanning a broader size and polarity range (Figure 7). All compounds have or are expected to have an ionization potential below 10 eV, allowing direct photoionization and/or a high proton affinity, allowing protonation by dopant assisted APPI through toluene used as solvent.^33–35^

**Figure 7:**
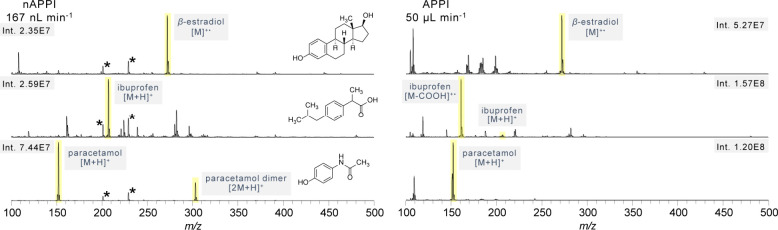
Spectra of different photoionizable analytes spanning a broader polarity range measured with APPI and nAPPI on a TSQ triple quadrupole MS. β-Estradiol and paracetamol were dissolved from neat compounds, and ibuprofen was measured from a pill. *Plasticizer impurities.

β-Estradiol, like other steroid molecules, has a relatively nonpolar character. In nAPPI, the radical molecular ion is formed, presumably by charge transfer from toluene ions. No fragments were recorded. In APPI the molecular ion of β-estradiol is also detected, however, large additional solvent ion attributed signals are also present.

Ibuprofen is a nonsteroidal anti-inflammatory drug with a structure that features both nonpolar and polar components, giving it an overall moderate polarity. Under nAPPI conditions ibuprofen shows protonation via a dopant-assisted ionization pathway. The analyzed sample was obtained by grinding a commercially available pill. The additional signals observed in the spectrum originate from the components of the pill. nAPPI exhibits significantly less fragmentation compared to APPI, resulting in a more intense molecular ion signal. With APPI the ibuprofen molecular ion was only detected with very low intensity; instead, ibuprofen undergoes fragmentation with the loss of a carboxylic group, producing the ion at *m*/*z* 161.

Paracetamol, a widely used analgesic, is a good example of pharmaceutical analytes, containing both polar (hydroxyl and amide) and nonpolar (aromatic) functionalities. In that case, the nAPPI spectrum also primarily shows protonation and the paracetamol dimer. APPI shows only the singly protonated molecule.

As an example for larger compounds, reserpine and gramicidin S were investigated with nAPPI. Both compounds are photoionizable.^36,37^ Reserpine is a plant-derived alkaloid and a well-known reference compound in mass spectrometry due to its stable and reproducible ionization properties. In APPI, the protonated reserpine signal exhibits higher intensity than the corresponding radical species. In contrast, with nAPPI, both species appear at nearly the same intensity, with the radical ion being slightly more prominent (Figure 8). In the mass range above 1000 Da, gramicidin S, a cyclic peptide antibiotic with amphiphilic properties, is ionized. For gramicidin S, the molecular ion is detectable using APPI, whereas nAPPI predominantly yields characteristic fragment ions form the loss of water.

**Figure 8:**
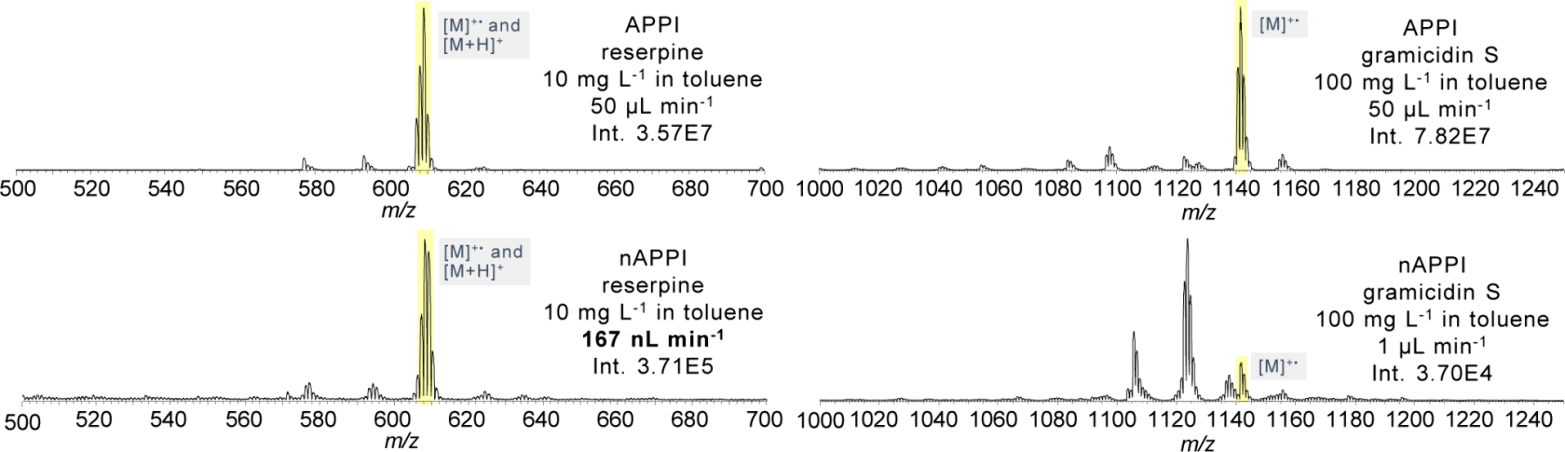
Two larger photoionizable analytes, reserpine (left) and gramicidin S (right) measured with APPI and nAPPI.

### nAPPI Performance with Real-World Samples

To investigate the performance of nAPPI with a highly complex real world sample, we compared measurements of a heavy crude oil using both APPI and nAPPI. Both spectra exhibit a bimodal distribution, extending up to the mass range of 800 Da, thus, providing comparable results despite the flow rate being reduced by a factor of 50 for the nAPPI measurement (Figure 9). This lower flow rate offers a considerable advantage for handling complex mixtures, as it reduces the likelihood of clogging and minimizes the need for time-intensive cleaning steps of the mass spectrometer.

**Figure 9:**
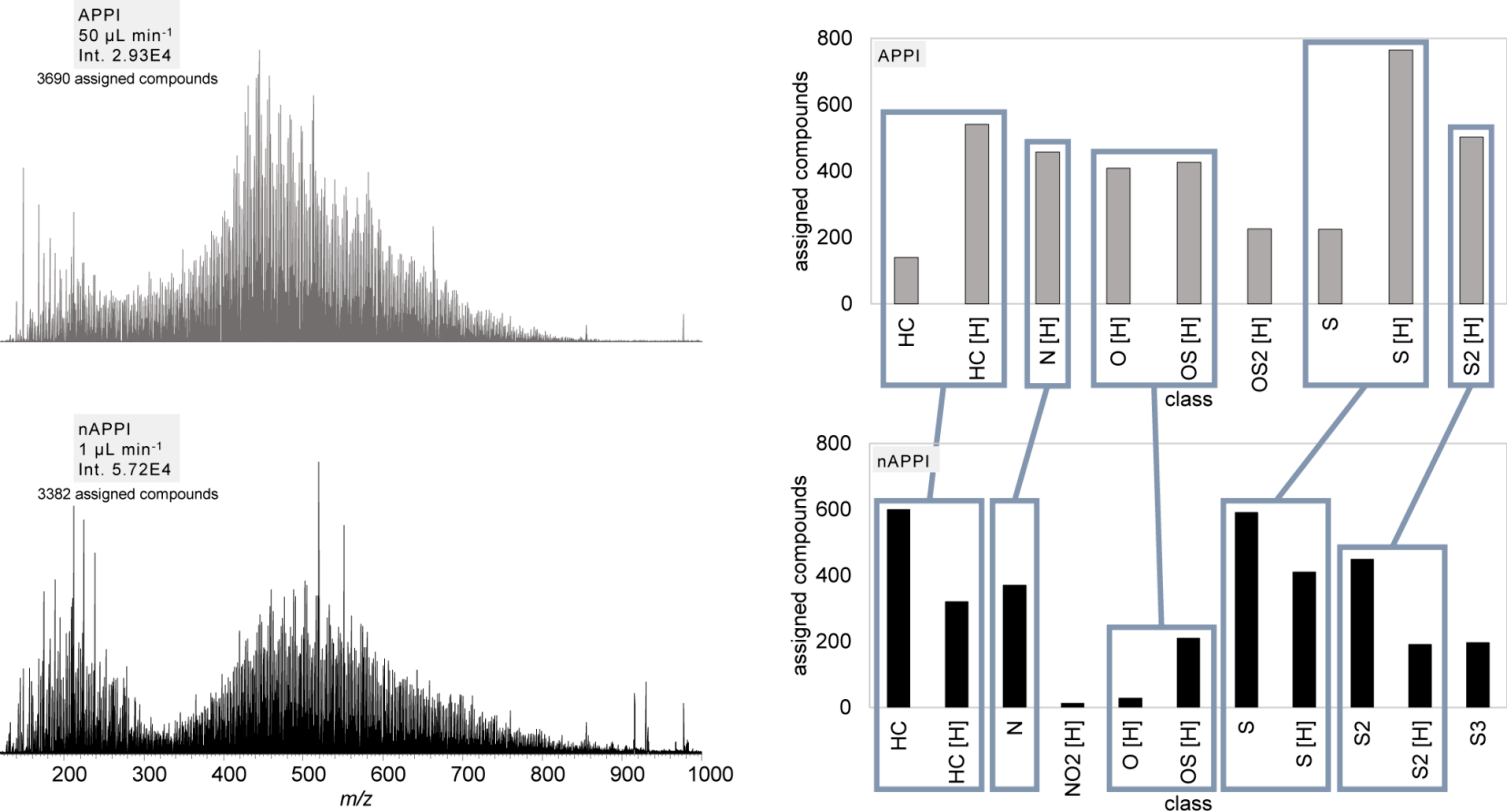
On the left: A complex sample of a heavy crude oil in toluene measured with conventional APPI and nAPPI. On the right: Assigned compositions of the heavy crude oil. While less oxygen-containing compounds are observed, there is an increased tendency for the formation of radical cations in nAPPI than in APPI.

While the results of both analyses are comparable in general, a detailed interpretation reveals differences between the methods. Figure 9 shows a plot of the heteroatom class distribution (number of heteroatoms per molecule) vs the number of elemental compositions found for a given class. Here, 'HC' denotes the hydrocarbon class (no heteroatoms) and '[H]' denotes compounds detected as protonated molecules instead of radical cations. A direct relation to specific compounds is mostly not possible in such analyses, as for each elemental composition, numerous different isomers are present in a crude oil that cannot be distinguished by mass spectrometry alone. Overall, the results show fewer oxygen-containing compounds detected with nAPPI than with APPI. Notably, the OS_2_ [H] class, containing about 200 compositions with APPI, was not observed with nAPPI. This is consistent with APPI being known for its ability to oxidize labile compounds, as for example sulfidic components but also aromatic compounds as toluene.^15^ The assumption that a large portion of the oxygenated compounds found with APPI originates from in-source oxidation is also backed by the high overlap with the corresponding oxygen-free classes (HC [H] and S_2_ [H], see Figure S7). Our results suggest that this behavior is far less prominent with nAPPI.

We attribute this mostly to the overall smaller setup that enables us to place the lamp directly beneath the MS orifice. Thus, there is almost no distance between the lamp window and the sample aerosol. This ultimately leads to less oxygen/water being present in the irradiated zone, minimizing the availability of oxidative species as has already been shown for toluene blank measurements.

The heteroatom classes NO_2_ [H] and S_3_ were uniquely observed in measurements using nAPPI, therefore no direct comparison is possible. A closer examination of the sulfur- and hydrocarbon-containing compound classes reveals distinct ionization behaviors: APPI primarily ionized compounds via proton transfer mechanisms, whereas nAPPI predominantly facilitated the formation of radical cations (Figure 9). Notably, in the S_2_ class, APPI did not generate any radical species, while nAPPI resulted in both radical and protonated ions. The protonation pathway appears to be less pronounced in nAPPI compared to conventional APPI. With pure toluene as the solvent, direct photoionization of the crude oil constituents can be assumed to be of negligible importance. Given the high toluene density in the sample plume, dopant-assisted APPI is at play in this case. Most analytes detected in a crude oil sample are made up of a more or less extended, substituted aromatic core and have an ionization potential below that of toluene. Ionization of most analytes by charge transfer reactions (eq 2) is therefore an expected outcome. Proton affinity is much more diversely distributed and hard to predict in such a complex sample. However, it can be anticipated that only a relatively small portion of the analytes can be protonated directly by toluene ions according to eq 3.^11^ Given that protonated water and/or solvent clusters are believed to be the main reaction partners involved in the generation of protonated analyte molecules (eqs 4+[Disp-formula eq5]), the relatively low abundance of protonated species is a strong indication that the availability of residual water within the ionization region is remarkably reduced compared to the conventional APPI setup. In total, there are 886 compositions that were detected as protonated species in APPI but only as radical species in nAPPI (24.0% (APPI) and 26.2% (nAPPI) of the total number of assigned compositions). The somewhat complementary behavior in ion formation is also depicted in a Kendrick-type plot in Figure 10 that shows all detected compositions for the S and the S [H] classes for APPI and nAPPI. This trend is also observed for the hydrocarbon class (Figure S8).

**Figure 10:**
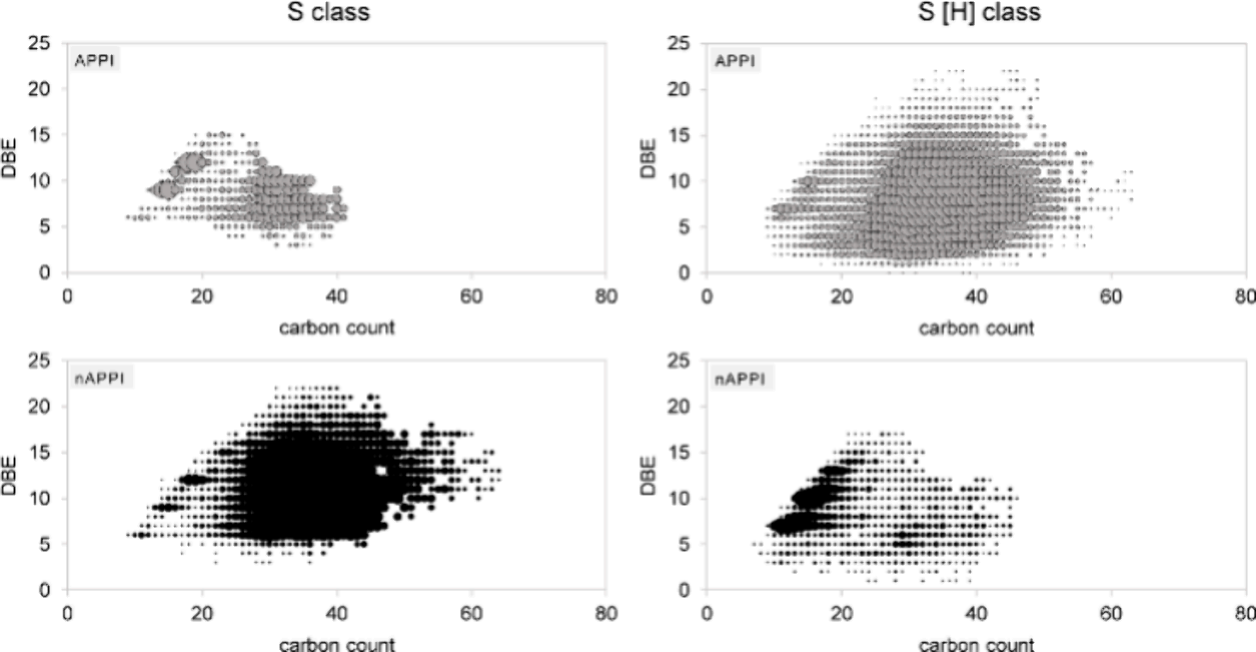
Kendrick plots of the S and S [H] class of the heavy crude for APPI and nAPPI.

The fact that an overall larger total number of compositions were assigned from the APPI measurements compared to nAPPI (654 more) is primarily due to oxygen-containing species. When considering all oxygen-containing species as a group, 832 compositions were assigned exclusively in the APPI measurement and were not detected by nAPPI. Only 24% of the total oxygen-containing compositions assigned for the APPI measurement were also found in the nAPPI data. Despite the differences in ion formation, the overall coverage of detected compositions is highly comparable, but in-source oxidation of compounds remains limited. Thus, the nAPPI source with its reduced flow rates presents a promising technique for analyzing complex, challenging samples.

## Conclusion

This study underscores the broader potential of miniaturized ionization techniques for photoionization mass spectrometry while maintaining or even surpassing the performance of conventional APPI. The idea was to maintain an optimal balance between solvent evaporation, analyte desolvation and ionization efficiency. With appropriate design modifications, nAPPI enables efficient ionization at nano flow rates while minimizing background noise and preserving high analytical performance resulting in improved sensitivity with higher a S/N ratio and lower LoD. This study demonstrates that both APPI and nAPPI sources exhibit similar functionality, with nAPPI successfully achieving miniaturization. Future work will focus on further optimization of key parameters to broaden the scope of real-world applications. Also, although our results indicate that the impact of residual water/oxygen in the ambient atmosphere is already reduced, spectra from toluene blanks indicated that elevated levels of oxygen are still present. Future works will therefore also focus on ways to further reduce this effect.

## Supplementary Material



## Notes

List of reference compounds and additional graphs highlighting the characteristics of the source (PDF)
