# Supplementary material for: Development of a Nanoscaled Ion Source for High-Sensitivity Photoionization Mass Spectrometry
Source: Anal Chem. 2026 Apr 10;98(15):11044–53. doi: 10.1021/acs.analchem.5c06912 (PMC13103928; doi:10.1021/acs.analchem.5c06912)
Supplement: Supplementary file 1 [file ac5c06912_si_001.pdf]

Supporting Information

Development of a Nanoscaled Ion Source for High-Sensitivity Photoionization Mass Spectrometry

Laura Tenhumberg, Wolfgang Schrader and Alessandro Vetere*

Max-Planck-Institut für Kohlenforschung, Kaiser-Wilhelm-Platz 1, 45470 Mülheim a. d. Ruhr

Contents

Figure S1 – Schematic comparison of source setup in APPI and nAPPI S3
Figure S2 – Temperature stability diagrams S3
Figure S3 – Power supply/electrode setup of miniaturized VUV lamp S4
Table S1 – Chemicals used in the study S4
Figure S4 – High-resolution mass spectrum of toluene S5
Figure S5 – Left: Long-term stability measurement of phenanthrene in toluene by nAPPI ... S5
Figure S6 – Signal intensity as a function of flow rate obtained using the nAPPI source S6
Figure S7 – Kendrick plots of the HC [H] and S_2 [H] classes of heavy crude oil S6
Figure S8 – Kendrick plots of the hydrocarbon classes of heavy crude oil S7

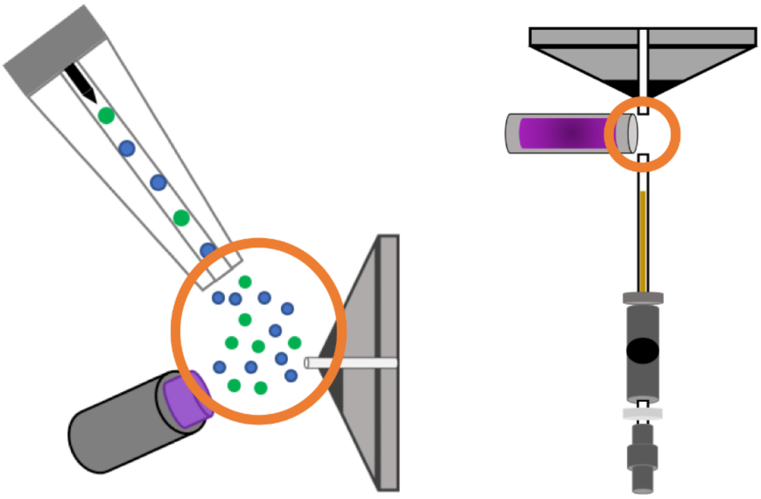

Figure S1. Schematic comparison of source setup and ionization zones in APPI and nAPPI. In APPI, the sprayer is angled at around 60° compared to the MS orifice and typically located at a distance of approximately 1.5-2.0 cm. The sprayer consists of an inner nozzle with a stream of nitrogen sheath gas applied to the tip to pneumatically spray eluting sample. The nozzle is slotted into a heated outer tube of 5.6 mm diameter, to which a supply of carrier gas (auxiliary gas, also N_2) is supplied. The VUV lamp (12.7 mm bulb diameter) is located on the side, perpendicular to the MS orifice and positioned 1.5 cm away from it. In comparison, the nAPPI design uses a smaller and simpler sprayer. No inner nozzle is used, but the sample capillary is introduced directly into the heated tube (1.65 mm diameter) with only a single gas supply present. The sprayer is mounted co-axially to the MS orifice at a distance of 6 mm. The VUV lamp (6 mm bulb diameter) is fitted directly onto the gap between MS orifice and sprayer outlet and the heated sprayer tube at a distance of approx. 1 mm.

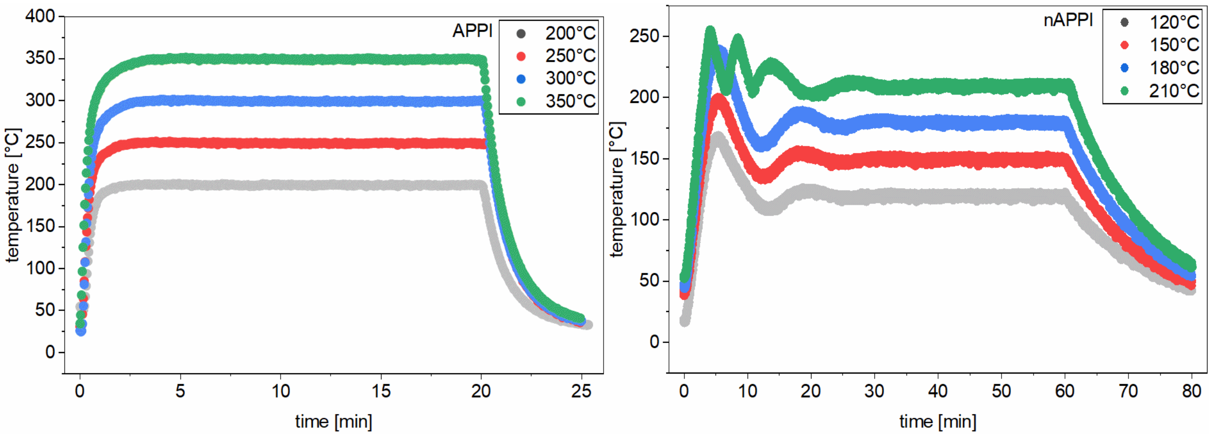

Figure S2. Heater readback for different API temperatures. The heating cartridge of is of 250 W for APPI (left) and of 200 W for nAPPI (right).

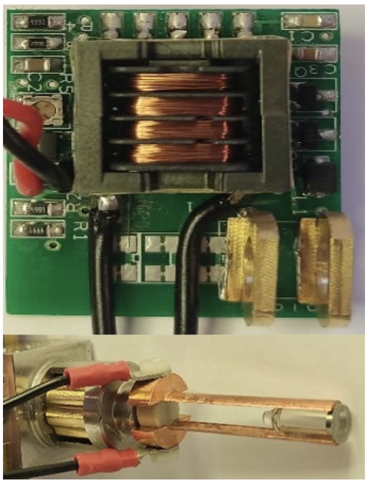

Figure S3. Power supply/electrode setup of miniaturized VUV lamp. Top: Original setup with two co-axial electrodes as supplied by Heraeus Noblelight GmbH. In this setup, the bulb is slotted through the electrodes. Bottom: The original electrodes have been disconnected from the circuitry and replaced by a pair of radially located electrodes that span a sector of 90° each. The electrodes are placed remote from the power supply PCB.

Table S1. Chemicals used in the study. *Compounds used for the PAH/PAXH mixture; **unknown.

chemicals	manufacturer	purity [%]	molecular weight [g mol^{-1}]
Benzo[a]pyrene*	Sigma-Aldrich	≥96	252.31
Carbazole*	Sigma-Aldrich	≥96	167.21
2-Cyclohexylethanobenzo-thiophene*	synthesized at MPI	**	244.40
Dibenzothiophene*	Sigma-Aldrich	98	184.26
17β-Estradiol	Sigma-Aldrich	≥98	272.39
Gramicidin S Hydrochloride from Bacillus brevis (Nagano)	Sigma-Aldrich		1141.47
Ibuprofen	from a pill	**	206.28
3-Methylquinoline*	Sigma-Aldrich	99	143.19
4'-Hydroxyacetanilid (Paracetamol)	Sigma-Aldrich	>98	151.16
Phenanthrene*	synthesized at MPI	**	178.23
Reserpine	Sigma-Aldrich	99	608.68
Heavy crude oil	**	N/A	N/A

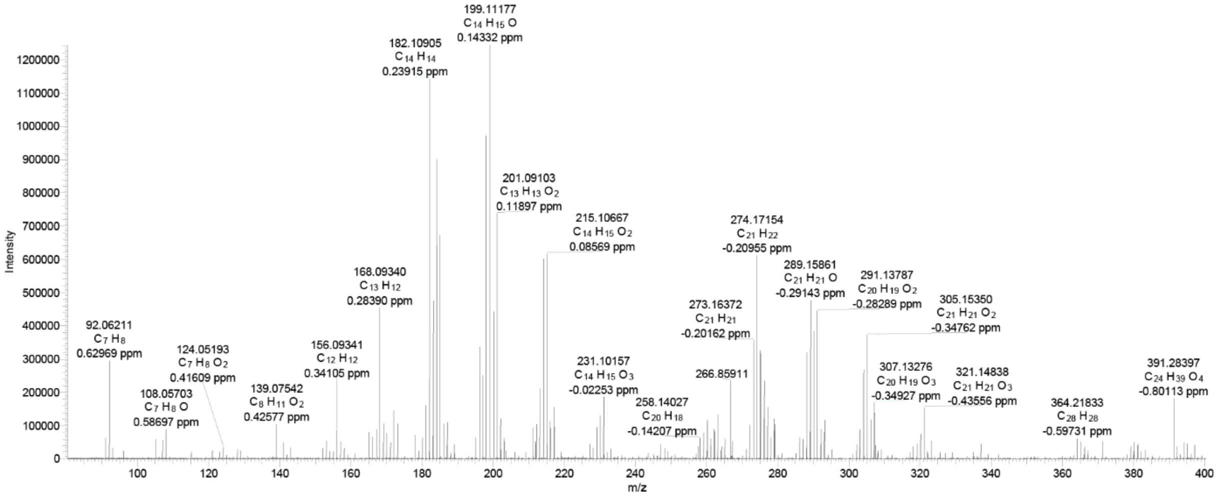

Figure S4. High-resolution mass spectrum of toluene measured with a conventional APPI source on an FT-ICR mass spectrometer (LTQ FT Ultra, 7 T, Thermo Scientific, Bremen, Germany). Assignments shown on most prominent signals are in good agreement with oxydation and/or oligomerization reactions of toluene occuring in the source region.

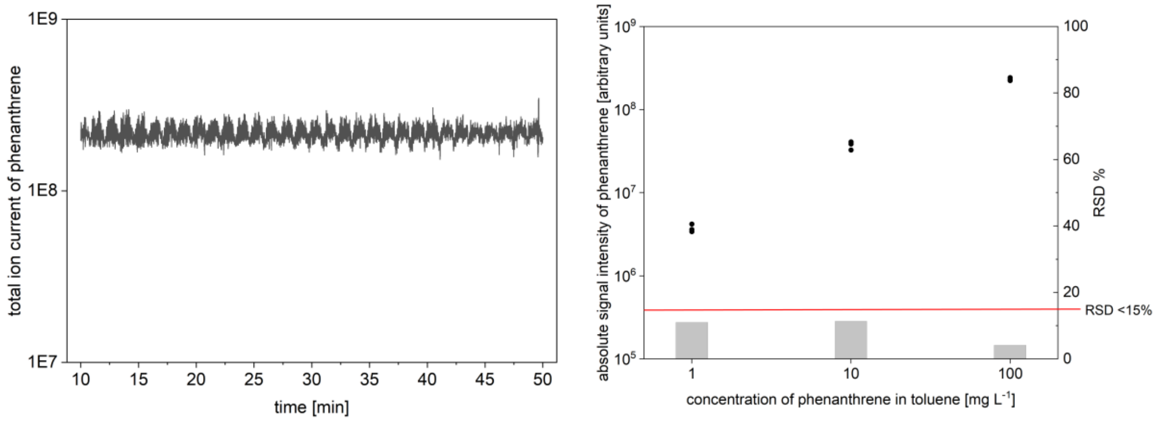

Figure S5. Left: Long-term stability measurement of phenanthrene in toluene by nAPPI, recorded at a flow rate of 1 µL min^{-1}. A high spray stability of RSD <10% even for nanoflow rates was observed. The temperature deviation is below 1.7 K independent from the desolvation temperature. The low frequency periodic fluctuations in the graph result from the syringe pump used for infusion. Right: Reproducibility of data obtained with the nAPPI source at different analyte concentrations. The data for each concentration were acquired on different days using a TSQ mass spectrometer. The data points represent the absolute signal intensity of the reference analyte phenanthrene on the left y-axis, while the bars indicate the corresponding relative standard deviation on the right y-axis. For all measurements, the RSD is below 15%.

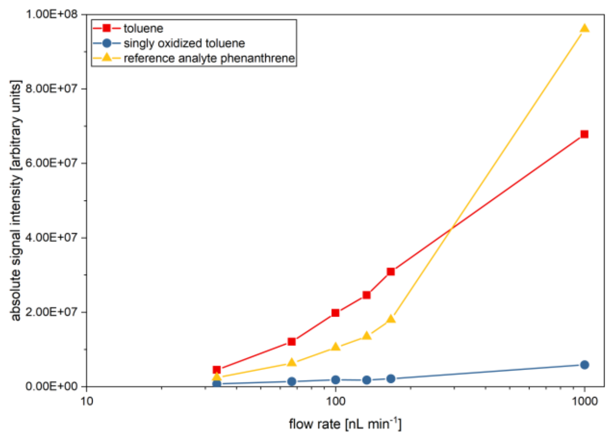

Figure S6. Absolute signal intensity as a function of flow rate obtained using the nAPPI source. The data are derived from mass spectra of the reference analyte phenanthrene in toluene. The three most intense signals from these spectra were used: toluene, singly oxidized toluene and phenanthrene.

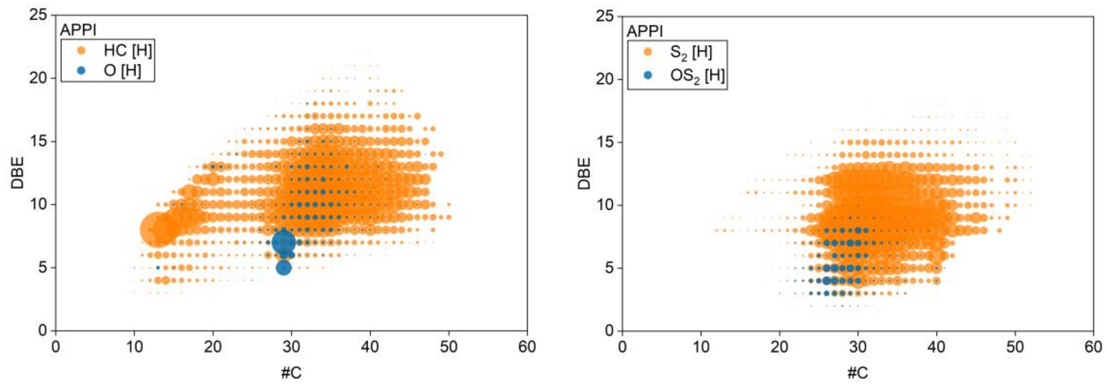

Figure S7. Kendrick plots of the HC [H] (left) and the S_2 [H] (right) classes (orange) of the heavy crude for APPI along with the corresponding classes that include one additional oxygen atom (blue, O [H] and OS_2 [H]). The oxygenated compounds largely follow the trend observed in the oxygen-free classes, indicating that in-source oxidation is an important side-reaction in APPI.

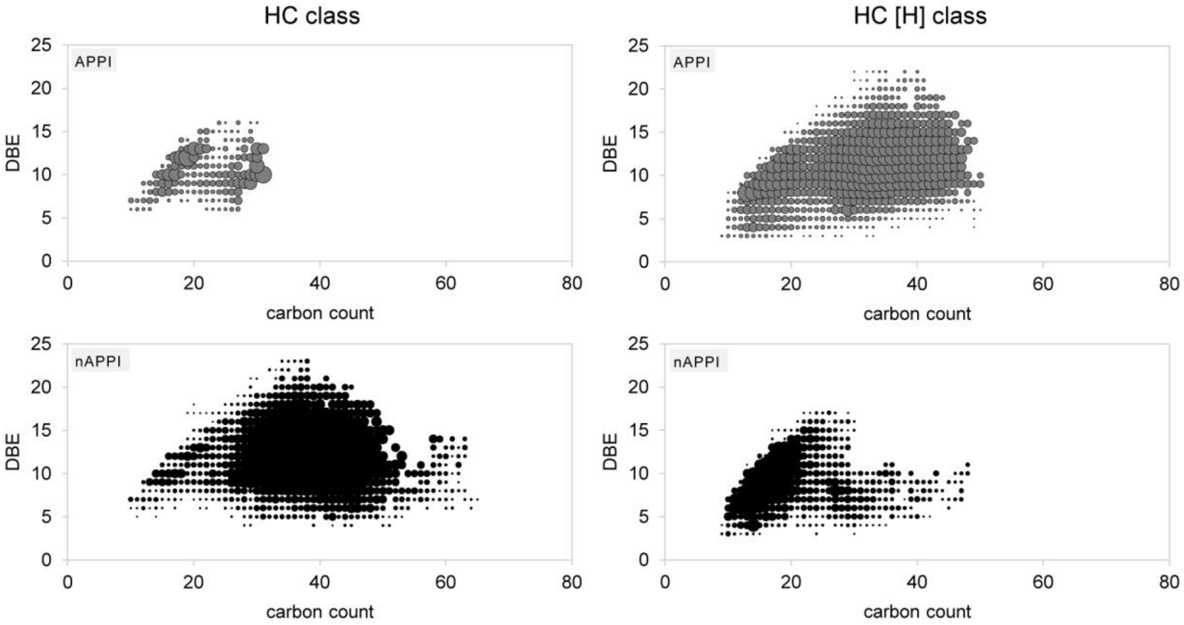

Figure S8. Kendrick plots of the HC and HC [H] class of the heavy crude for APPI and nAPPI.
